# High-Precision Radiosurgical Dose Delivery by Interlaced Microbeam Arrays of High-Flux Low-Energy Synchrotron X-Rays

**DOI:** 10.1371/journal.pone.0009028

**Published:** 2010-02-03

**Authors:** Raphaël Serduc, Elke Bräuer-Krisch, Erik A. Siegbahn, Audrey Bouchet, Benoit Pouyatos, Romain Carron, Nicolas Pannetier, Luc Renaud, Gilles Berruyer, Christian Nemoz, Thierry Brochard, Chantal Rémy, Emmanuel L. Barbier, Alberto Bravin, Géraldine Le Duc, Antoine Depaulis, François Estève, Jean A. Laissue

**Affiliations:** 1 Université de Toulouse, UPS, Centre de Recherche Cerveau et Cognition, Toulouse, France; 2 CNRS, CerCo, Toulouse, France; 3 ESRF, Grenoble, France; 4 Department of Medical Physics, Stockholm, Sweden; 5 Inserm, U836, Grenoble, France; 6 Université Joseph Fourier, Grenoble Institut des Neurosciences, UMR-S836, Grenoble, France; 7 Department of Neurosurgery, CHU de Grenoble, Grenoble, France; 8 Institute of Pathology, University of Bern, Bern, Switzerland; The University of Chicago, United States of America

## Abstract

Microbeam Radiation Therapy (MRT) is a preclinical form of radiosurgery dedicated to brain tumor treatment. It uses micrometer-wide synchrotron-generated X-ray beams on the basis of spatial beam fractionation. Due to the radioresistance of normal brain vasculature to MRT, a continuous blood supply can be maintained which would in part explain the surprising tolerance of normal tissues to very high radiation doses (hundreds of Gy). Based on this well described normal tissue sparing effect of microplanar beams, we developed a new irradiation geometry which allows the delivery of a high uniform dose deposition at a given brain target whereas surrounding normal tissues are irradiated by well tolerated parallel microbeams only. Normal rat brains were exposed to 4 focally interlaced arrays of 10 microplanar beams (52 µm wide, spaced 200 µm on-center, 50 to 350 keV in energy range), targeted from 4 different ports, with a peak entrance dose of 200Gy each, to deliver an homogenous dose to a target volume of 7 mm^3^ in the caudate nucleus. Magnetic resonance imaging follow-up of rats showed a highly localized increase in blood vessel permeability, starting 1 week after irradiation. Contrast agent diffusion was confined to the target volume and was still observed 1 month after irradiation, along with histopathological changes, including damaged blood vessels. No changes in vessel permeability were detected in the normal brain tissue surrounding the target. The interlacing radiation-induced reduction of spontaneous seizures of epileptic rats illustrated the potential pre-clinical applications of this new irradiation geometry. Finally, Monte Carlo simulations performed on a human-sized head phantom suggested that synchrotron photons can be used for human radiosurgical applications. Our data show that interlaced microbeam irradiation allows a high homogeneous dose deposition in a brain target and leads to a confined tissue necrosis while sparing surrounding tissues. The use of synchrotron-generated X-rays enables delivery of high doses for destruction of small focal regions in human brains, with sharper dose fall-offs than those described in any other conventional radiation therapy.

## Introduction

Dose delivery in brain radiosurgery is limited by the tolerance of normal tissues surrounding the target. In some particular cases, the dose gradients (from the irradiated target to surrounding sensitive structures) achievable with high-energy photons (MeV) do not allow curative doses to be delivered at the target without injuring adjacent functional structures [1], [2]. Brain treatment research based on kilovoltage-photons was discontinued when Leksell introduced the radiosurgery technique in the early 50′s [3]. Nowadays, kilovoltage X-rays are considered as inefficient for most clinical applications by radiotherapists because of their low penetrative capability. Indeed their use is limited to treatments of superficial diseases.

Here we show the potential applicability of low-energy photons for irradiation of small circumscribed brain regions with sub-millimeter precision. Microbeam Radiation Therapy (MRT) [4], [5], an innovative radiosurgical technique, takes advantage of the particular properties of synchrotron generated X-rays (50–350 keV) to deliver very high doses (several hundreds of Gy) using arrays of spatially distributed quasi-parallel microplanar beams (MBs) (25–75 µm wide and spaced 100–400 µm on-center). The fundamental phenomenon of the large dose-volume effect at a microscopic scale, firstly described by Zeman and Curtis in the 60′s [6]–[8], can presently be performed only at 3^rd^ generation synchrotron sources. Those can provide an adequate dose rate, energy spectrum and a minimal beam divergence, allowing the deposition of the steep dose gradients between MBs. The strong radioresistance of normal brain vasculature [9], [10] to spatially fractionated exposures to MBs may prevent the decrease in brain perfusion and hypoxia observed after conventional radiotherapy [11], [12]. Further, by interlacing arrays of MBs and delivering them to a target via several ports [13], normal tissues surrounding the target will be exposed to a well tolerated, spatially fractionated dose, whereas the radiation target will receive a high homogenous radiation dose.

In this study, we show that it is possible to induce a focal damage in the caudate nucleus of normal rat brains by interlacing 4 arrays of 10 MBs separated by a 45° rotation angle. The 7 mm^3^ lesion and the surrounding tissues were observed up to one month using MRI and immunohistopathology. As a proof of principle for the relevance of the method, we used interlaced arrays of microplanar beams (IAMB) to treat seizures in the Genetic Absence Epilepsy Rats from Strasbourg (GAERS) [14], [15]. A complementary dosimetry study using a human-sized head phantom highlights advantages for the potential use of synchrotron low-energy photons for clinical brain radiosurgery.

## Methods

All operative procedures related to animal care strictly conformed to the Guidelines of the French Government (licenses 380324 and A3818510002). Experiments were performed under anesthesia, 5% isoflurane for induction, intraperitoneal injections of xylazine/ketamine for irradiations (64.5/5.4 mg.kg^−1^) and 2.5% isoflurane for MRI follow-up were used for maintenance.

### Radiation Source

IAMB irradiations were performed on the ID17 biomedical beamline at the European Synchrotron Radiation Facility (Grenoble, France) [16]. IAMB uses X-rays emitted tangentially from relativistic electron bunches circulating in a storage ring. The wiggler source (a magnetic structure of alternating poles positioned on a straight section of the storage ring) produces a wide spectrum of photons which extends, after filtration (Be (0.5 mm), C (1.5 mm), Al (1.5 mm) and Cu (1.0 mm) from 50 over 350 keV (median energy: 107 keV). The quasi-laminar beam was collimated into an array of rectangular MBs of 52 µm×2 mm (width x height) with 2 pairs of slits positioned about 42 m from the photon source and 1 m upstream from the head of the animals. The dose rate in air at the animal surface was approximately 16 000 Gy.s^−1^.

### Irradiation Geometry (Right Caudate Irradiation)

**Figure 1:**
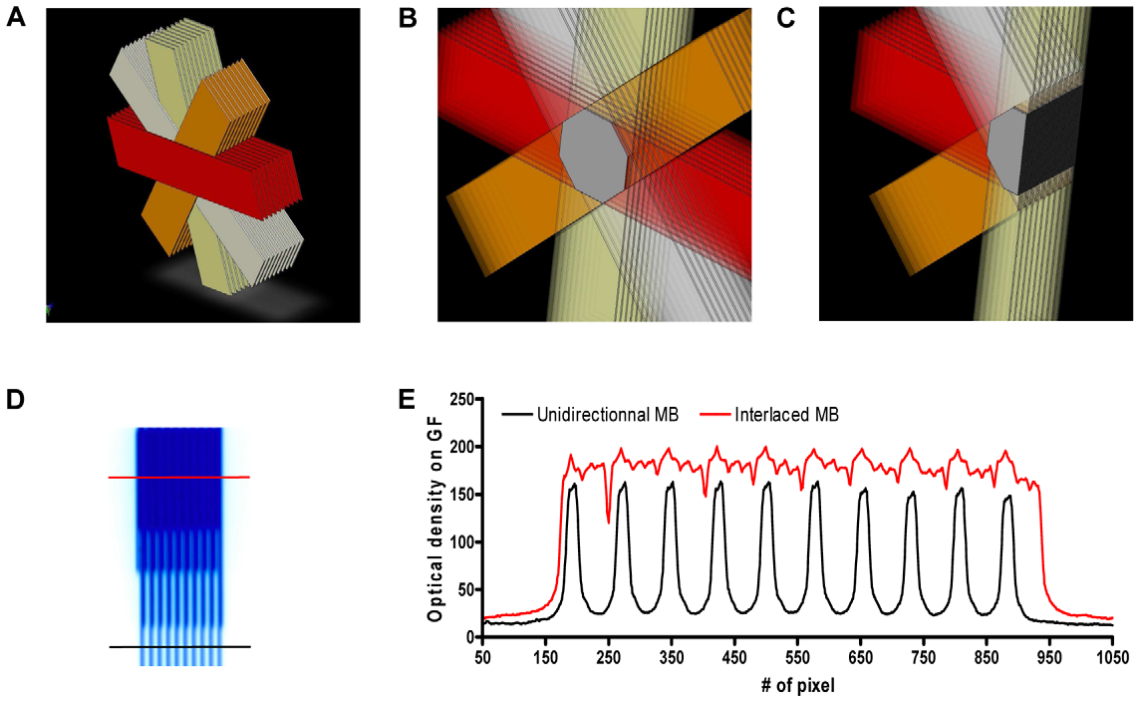
Schematic representation of the irradiation geometry in normal rats. (A–C). Four arrays of 10 MBs (50 µm wide, 200 µm on-center distance) were interlaced and created a 2×2×2.2 mm^3^ target region where the radiation dose is homogenous. D- Gafchromic® film image of interlaced MBs; the upper part corresponds to a centre-to-centre distance of 200 µm. The radiation target corresponds to the region where all the 4 arrays of MBs interlaced. E- Dose profiles measured on the Gafchromic® film shown in (D). The red line shows the dose in the interlaced region. The dose profile produced in the spatially fractionated irradiation and which is delivered by a single array of MBs is shown with the black line.

The animals were fixed by the teeth in a vertical position, on a home made Plexiglas frame and placed in front of the X-rays source on a Kappa-type goniometer (Huber, Germany), with which the rat can be translated and rotated. Stereotaxic coordinates of the “radiation target”, *i.e*., the tissue volume in which the 4 arrays interlaced, were measured on MRI images based on external marks of the animal head. The irradiation geometry is depicted on Fig. 1. The center of the target was located in the right caudate nucleus, at the intersection of 3 planes: a) 10 mm posterior to a coronal plane centered externally on both eyes; b) 3 mm to the right of the mid-sagittal plane, and c) 6.8 mm below a horizontal plane on the top of the rat's head. Each port of irradiation was composed of 10 MBs, 52 µm wide, spaced 200 µm on-center. The first irradiation exposure was performed after an axial −45° rotation around the center of the target volume (position −45°). The rats were then replaced to the initial position (position 0°) by a 45° rotation and moved down by 50 µm before the second exposure. This cycle was repeated twice for the exposures at positions +45° and +90°, respectively. The 50 µm z-step applied between each irradiation produced a 7 mm^3^ target volume in which all MBs were interlaced. The in-microbeam entrance dose was fixed at 200 Gy. Uniformity and the size of the MBs were checked with the help of Gafchromic® films. The incoming spatially non-fractionated dose was measured using an ionization chamber and the mid-valley doses were calculated with Monte Carlo (MC) simulations.

As a control group, we irradiated 4 rats with 4 intersecting (non-interlaced) MBs arrays (2 mm high, 52 µm wide and spaced 200 µm center-to-center) with an angle of 45° between each array. According to MC simulations, the resulting peak dose at the intersection site (right caudate nucleus, 1 cm depth) was 700 Gy for a skin entrance dose of 200 Gy delivered in each port.

### Monte Carlo Simulations and Dose Calculations

The 2006 version of the MC code PENELOPE was used to calculate the radiation dose distributions inside a rat, as well as a human-head phantom [17]. Both phantoms were considered to be made entirely of water. The same code has also been used in earlier studies to characterize the doses used in MRT [18]. PENELOPE is a Monte Carlo code, with which it is possible to simulate the coupled photon and electron transport through arbitrary amorphous media. The most common electron and photon interactions are accounted for as well as the production of secondary particles with a kinetic energy above a certain creation threshold. The paths and energy losses of the X-rays and the secondary electrons they generate were in this work followed down to a cutoff energy of 1 keV. For the X-ray spectrum used in MRT (mean energy ∼107 keV) the most common interaction type in tissue-like materials is Compton scattering. The so called mixed simulation parameters for electron scattering in PENELOPE were set to small values (C1 = C2 = 0.01) to make sure that the program is running mainly in the so-called detailed simulation mode, where all electron collisions are handled individually, no matter how small the collision/deflection is.

In this work, the dose distribution produced by a single, rectangular (“planar”) x-ray microbeam was simulated inside the head phantoms which had a cylindrical shape. The X-rays were set to start on top of the cylinder surface with a direction parallel with the cylinder axis. The X-ray energies of the incident beam were sampled from the spectrum measured at the ESRF. Since the divergence of the X-ray beams produced at synchrotrons is small, the microbeams were considered to be perfectly parallel in the simulations and incident perpendicular to the phantom surface. Doses were scored at different depths in the phantom, in volume elements (voxels) with the shape of parallelepipeds [e.g. inside the microbeam the volume element size was 1 µm (transverse direction) × the microbeam height ×1 mm (the element length in the depth direction). Further away from the center of the microbeam, in the transverse direction, the voxel size was increased to obtain good statistics. A transversal dose profile for a chosen depth was then prepared from the simulation output. Using the dose profile for a single microbeam, the composite dose distributions for the same number of microbeams as was used experimentally was obtained by using an addition/superposition procedure described in Siegbahn et al. [19]. Finally, the positions of the centermost peak and valley doses in the composite dose distribution for the microbeam array were located and the so called PVDR (Peak-to-Valley Dose Ratio) was evaluated.

#### Monte Carlo simulation and dose calculations in the rat brain

The rat head was simulated by a water cylinder of 3 cm height and of 1.5 cm radius. A microbeam field size of 50 µm (width) ×2 mm (height) was used in the simulations. Each microbeam array contained 10 microbeams and therefore 40 microbeams were interlacing in the irradiation target. For the calculation of the dose distribution produced in the rat head by IAMB, it was assumed that the distance from the entrance point for all four microbeam arrays (at the rat head surface) to the point of cross-firing was 1 cm. The four microbeams arrays then produced an identical dose distribution at 1 cm depth, independent of the angle of incidence. In that way, the resulting dose distribution in the cross-fired volume, when using the interlaced technique, was obtained by adding the dose profile at 1 cm depth for a single microbeam 40 times, with an incremental shift of 50 microns for each added microbeam. For the control group with 700 Gy (non-interlaced) target dose, the same relative dose distribution as that calculated for a single microbeam array was used.

#### Monte Carlo simulations and dose calculations in a human-sized head phantom

The human head was simulated by a water cylinder of 16 cm height and of 8 cm radius. In this case the simulations were done for three different array sizes: 2×2 mm^2^, 1×1 cm^2^ and 3×3 cm^2^. The latter two array sizes were believed to be of potential interest to treat tumors in humans. The simulations and calculations were then done in analogy to those done for the rat head. More detailed calculations were done in the human head phantom of the variation of the peak and valley doses with depth.

### Magnetic Resonance Imaging

MRI experiments were performed at 7T (Bruker Avance III system) using a quadrature volume coil. After anesthesia, a catheter filled with heparinized saline was inserted into the dorsal tail vein of the animal. Three to eight rat brains were imaged at different delays after exposure *i.e.* 1, 4, 7, 15 and 30 days. Radiation-induced anatomical changes were assessed on T_2_-weighted images (RARE sequence. TR: 5 s, effective TE: 33 ms, FOV: 3×3 cm^2^, matrix: 64×64, 0.5 mm-thick). The apparent diffusion coefficient (ADC) of water was mapped from day 4 to day 30 after irradiation using a diffusion tensor MR sequence (DTI-EPI, TR: 2.5 s, TE: 30 ms, FOV: 3×3 cm^2^, matrix: 64×64, 1 mm-thick, 3 reference images and 6 directions with b = 1000 s.mm^−2^). Brain vessel permeability was then characterized using a T_1_-weighted MR sequence (RARE sequence. TR: 950 ms, effective TE: 7.7 ms, FOV: 3×3 cm^2^, matrix: 64×64, 0.5 mm-thick) acquired 5 min after an intravenous injection of Gd-DOTA (200 µmol.kg^−1^). Two regions of interest were defined: the radiation target in right caudate and the contralateral left caudate nucleus. A two way ANOVA test (Bonferroni posttest) was used to compare data across groups (*: p<0.05, **: p<0.01, ***: p<0.001).

### Immuno/Histological Analyses

One, 7, 14, and 30 days after IAMB, four rats were randomly chosen and killed by Dolethal overdose. Twenty µm thin coronal brain sections were cut at −20°C on a cryostat (Microm HM560, France) and stained with hematoxylin/eosin. For immunohistology, sections were fixed with PFA 4% for 15 min and blocked with donkey normal serum (DNS, Interchim) diluted in phosphate-buffered saline (PBS) 1X for one hour (PBS/DNS 5%). For neuronal staining, the sections were pretreated with alcohol 50% in PBS for 1 h at room temperature, then incubated with the primary antibody (NeuN, 1/1000, Chemicon) in PBS-Triton 0.3% for 48 h.Other immunolabelings were performed as previously described [20]. Primary antibodies used included: Glial fibrillary acidic protein (1/2000, anti-GFAP, Z0334 DakoCytomation); Collagen IV (1/800, F-5202 VF83, UNLD); ED1: (1/2000, AbC117-6714, AbCys); pH2AX (1/500, 05636, Upstate); Ki67 (1/200, Clone S6, Lab Vision Corporation, Fremont) diluted in PBS/NDS 1%. Sections were washed 4 times with PBS, then exposed to the secondary antibodies Alexa Fluor-conjugated donkey F(ab')2 (1/200, # A31571, A11056, A11055, A10040, A21206, Invitrogen) for 2 h at room temperature and nuclei were counterstained by DAPI (1 µg.ml^−1^ in mounting medium). The sections were examined with a Nikon Eclipse E600 microscope equipped for epifluorescence with x10 and 20 X objectives.

### Bilateral IAMB Irradiations of Somatosensory Cortex in the GAERS Rat

As a proof of concept, bilateral IAMB irradiation of the somatosensory cortex, shown to initiate spontaneous epileptic seizures, were performed in the GAERS [14], [15]. Rats were irradiated with a 200 Gy entrance dose using two different MB arrays which generated two contiguous radiation targets (4.1 and 2.7 mm long, 1.5 mm high). Ten days after irradiation, anesthetized GAERS were placed into a stereotaxic frame. Three stainless steel wire bipolar electrodes were implanted in the S1 somatosensory cortex (anteroposterior (AP), 0 mm; medio- lateral (ML), 4.6 mm; dorsoventral (DV), −3.0 mm), in the motor cortex (AP, 3.2 mm; ML, 2.0 mm; DV, −2.0 mm) and in the ventral posteromedian nucleus of the thalamus (AP, 2.5 mm; ML 2.7 mm; DV −5.4 mm) with the bregma as the reference. A reference electrode was placed over the cerebellum. All electrodes connected to a female connector. Six electroencephalograms (EEG) were recorded (System Plus, Micromed France SAS) on freely moving rats between D18 and D55 after exposure. We report the cumulated duration (min) of the 7–9 Hz spike waves discharges monitored per hour in control (unirradiated) and irradiated groups.

## Results

### Post-Irradiation Animal Behavior

All rats irradiated in the right caudate nucleus survived the observation period of 1 month without any sign of neurological disorder; the evolution of their body weight was recorded as normal (data not shown). A slight hair loss was observed at the entrance site of each of the 4 different arrays of MBs between 14 days and 1 month after irradiation.

### Normal Rat Dosimetry

The irradiation geometry and experimental dosimetry for rats irradiated in the right caudate nucleus are shown in Fig. 1. As shown on the gafchromic® film in Fig. 1D, the 4 arrays of 50 µm wide MBs interlaced correctly in the radiation target. The dose profile (Fig. 1E) read by optical density on the gafchromic® film after exposure revealed that the quasi-homogenous radiation dose measured in the interlaced region was higher than the one measured in the MBs path. Monte Carlo simulations showed that the dose in the radiation target was 200 Gy, while the in-MBs dose at 1 cm depth would be of 175 Gy. The corresponding valley dose in the surrounding tissues was 3.1 Gy (PVDR∼56).

### MRI Follow-Up of the Radiation-Induced Cerebral Lesion

**Figure 2:**
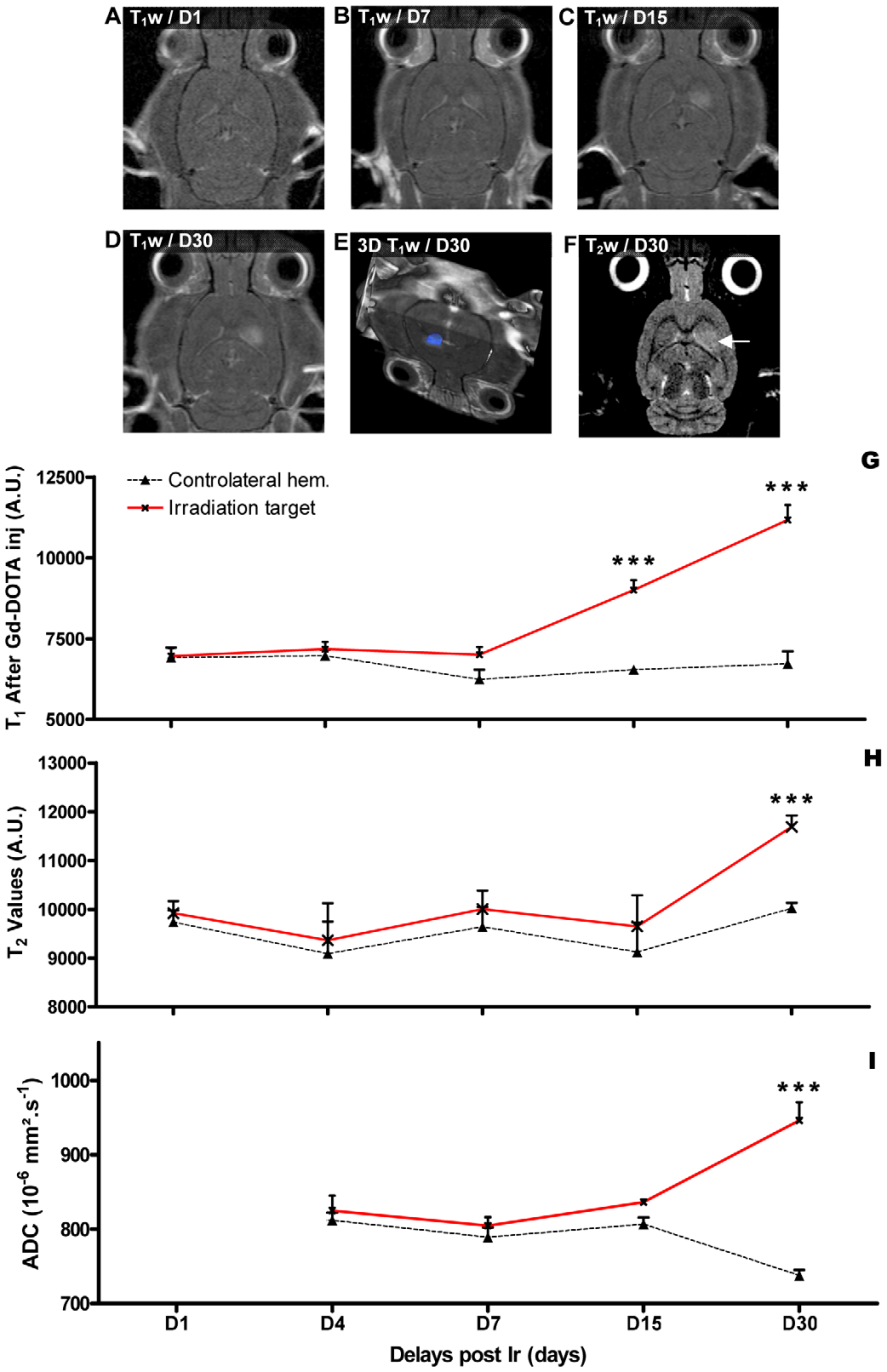
Temporal MRI follow up of the radiation target. A-D- MR characterization (T_1_-weighted images 5 min after Gd-DOTA injection) of the evolution of the radio-induced lesion between D1 and D30 after exposure. E-Three-dimensional reconstruction of the irradiated target (blue) based on Gd-DOTA extravasation on T_1_-weighted images at D30 after exposure. F-T_2_-weighted MR images acquired irradiation and reflecting brain edema in the radiation target indicated by a white arrow. G-I Evolution of the T_1_, T_2_ (arbitrary values) and ADC values measured at different delays after irradiation in the radiation target (red lines) and in the contralateral hemisphere (black lines). ***: significantly different from time matched control (p<0.001).

The evolution of the brain lesions in the rats with IAMB irradiation of the right caudate nucleus was imaged by MRI one, 4, 7, 15 and 30 days after irradiation (Fig. 2). No changes were observed between 24 h and 4 days after irradiation. There was no significant difference in T_1_w and T_2_w values between the irradiated target and the contralateral hemisphere (Fig. 2G–H). At D7 after exposure, 3/7 rats exhibited Gd-DOTA extravasation in the radiation target (Fig. 2B). T_1_w values became significantly higher in the radiation target at D15 after IAMB irradiation, when 8/8 rats exhibited Gd-DOTA diffusion in the brain parenchyma (Fig. 2C). However, there was no time-matched modification of ADC and T_2_w values (Fig. 2I). At D30 after interlaced MRT, 8/8 rats showed a highly localized extravasation of Gd-DOTA and T_1_w values were higher than the T_1_w at D15 (Fig. 2D–E–G). These changes were correlated with a significant increase in ADC and T_2_w values (Fig. 2H–I). T_1_w and T_2_w hypersignals were detectable on 4 to 5 MR slices (Fig. 2F), *i.e.,* on a vertical distance of ∼2.25 mm. A three-dimensional representation of the radiation-induced lesion is shown in Fig. 2E.

**Figure 3:**
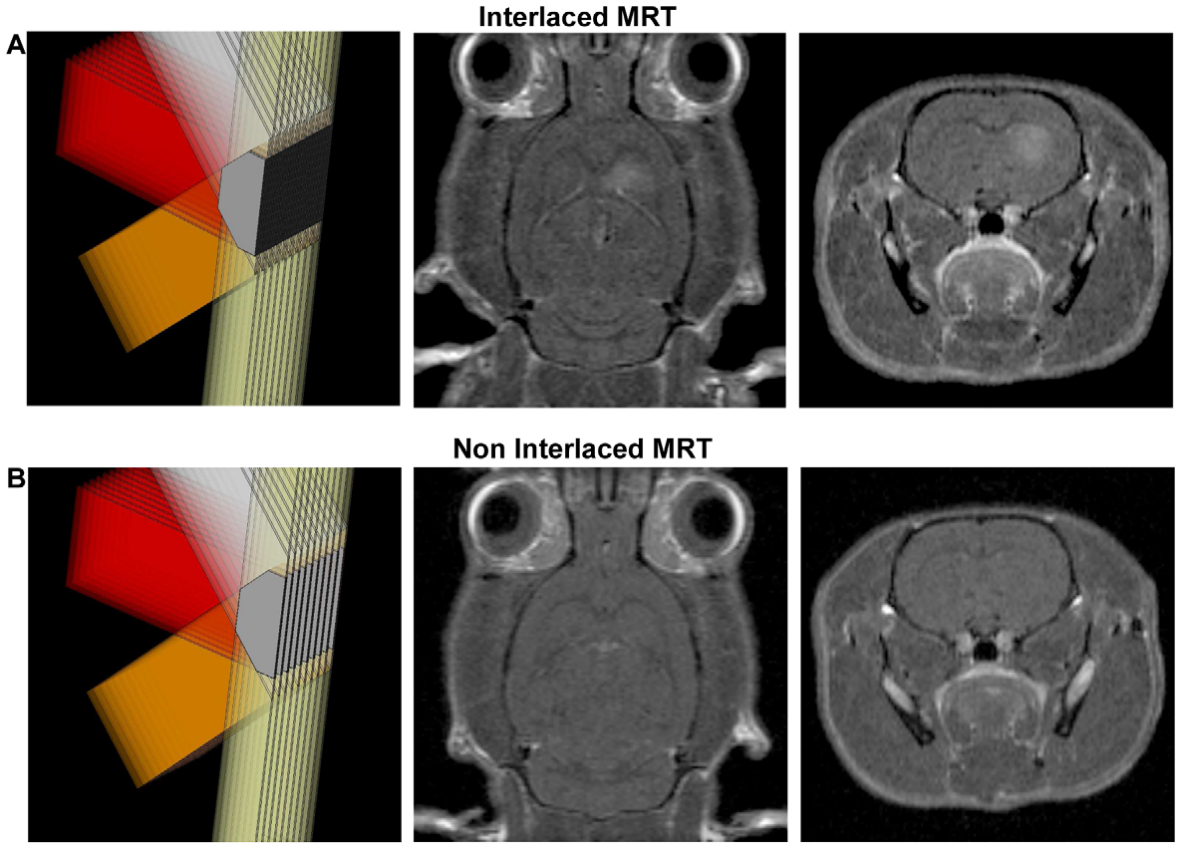
Effects of interlaced vs. non-interlaced microbeam irradiations on normal brains. MR transverse and coronal T_1_w images (5 min after Gd-DOTA i.v. injection) acquired 30 days after interlaced (A) and non interlaced MRT (B). The extravasation of the constrast agent is only detectable after interlaced MRT, the 700 Gy peak dose, resulting from superimposed but non interlaced MRT does not induce Gd-DOTA extravasation in the brain parenchyma.

The rats in the control groups (identical, but non-interlacing arrays) received an 700 Gy peak dose in the MB paths in the tissue volume delimited by the intersection of the 4 arrays. The calculated mid-valley dose was 12.5 Gy. The rat brains were imaged at D30, *e.g.* when the IAMB irradiation induced the most important brain changes in rats as detected by MRI. No extravasation of Gd-DOTA or modification in T_1_w and T_2_w values were found in those control animals exposed to non-interlacing arrays (Fig. 3).

### (Immuno)histological Analysis of the Cerebral Lesion Evolution

**Figure 4:**
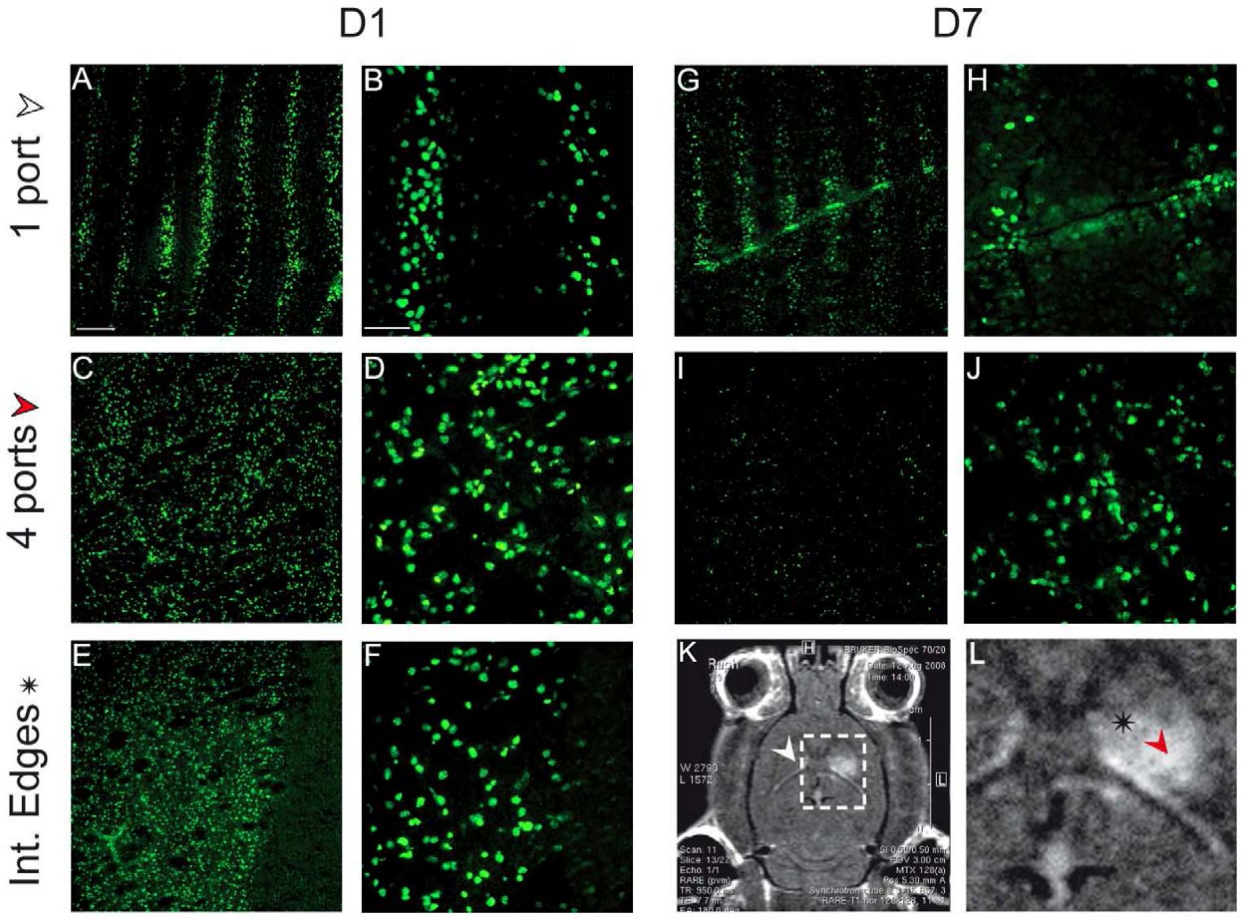
Immunohistological verification of the irradiation geometry. pH2AX immunolabeling performed (DNA damages) at D1 (A–F) and D7 (F–J) in different brain regions reported on MR-images (K,L). The first row corresponds to the contralateral hemisphere (1 port), the second one the radiation target (4 ports) and the last one to the edges of the radiation target. Scale bars represent: 200 µm (A, C, E, G, I) and 50 µm (B, D, F, H, I).

**Figure 5:**
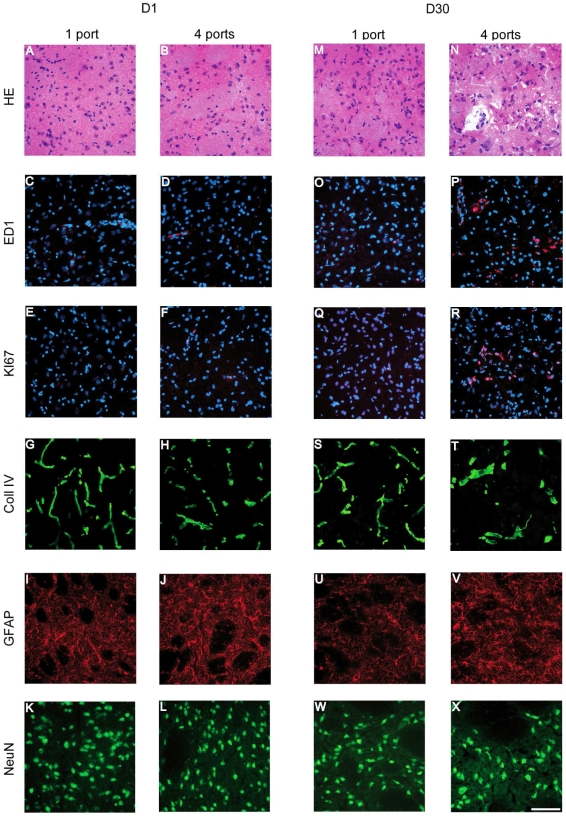
Temporal immunohistological follow up of the radiation target. (Immuno) histological study of the contralateral hemisphere (1 port) and the radiation target (4 ports) at D1 (A–L) and D30 (L–X) after irradiation. The different rows correspond to HE staining, monocyte/macrophage labeling (ED1, red labeling, nuclei counterstained with DAPI), cycling cells (Ki67 positive cells, red labeling, nuclei counterstained with DAPI), brain vessels (type-IV collagen), astrocytes (GFAP) and neurons (NeuN). Scale bar represents 200 µm.

The paths of MBs and signs of radiation induced-DNA damage were evident in γH2AX immuno-labeled sections; ionizing radiation induces DNA double strand breaks which were linearly correlated with the number of gamma-H2AX foci within cell nuclei [21]. Figure 4A–B shows the irradiation pattern, observed 24 h after exposure, in the left hemisphere, opposite to the radiation target, *i.e*., approximately 50 µm-wide tracks spaced about 200 µm on-center. Conversely, the right caudate nucleus, exposed to the 4 interlaced arrays, exhibited a quasi-uniform pH2AX labeling (Fig. 4C–D). One day after irradiation, cellular details and the paths of the MBs were not identifiable on sections stained with hematoxlin and eosin (Fig. 5A–B). The number of gamma-H2AX foci in both locations decreased with time after irradiation. Few of them remained detectable at D7 (Fig. 5E–F) and no cell nucleus was labeled from D15 to D30 after exposure. However, on D14, small (up to 1 mm diameter) perivascular tissue zones in the left caudate nucleus displayed loss of cohesion, presence of few nuclear fragments, hypocellularity, eosinophilia, and minute focal hemorrhage on sections stained with hematoxylin and eosin. Few polymorphonuclear granulocytes and mononuclear cells were present in such areas. On D30, the left caudate nucleus appeared larger than the right one, displaying areas up to 1.5 mm in diameter with lack of normal tissue cohesion, hypocellularity, diminished eosinophilia, many interstitial microvacuoles and prominent perivascular Virchow-Robin spaces (Fig. 5M–N).

Immunohistochemistry for ED1 showed a time-related increase of macrophages/monocytes number in the radiation target. The maximal number of macrophages/monocytes in the interlaced region was detected at D30 after exposure. On the contrary, the spatially fractionated, non-interlaced irradiation mode did not induce such cellular responses in the left, contralateral hemisphere (Fig. 5C–D, O–P). An analogous labeling pattern was observed for the Ki67 antibody: immunoreactive cells were only detected in the radiation target; they reached a maximum 1 month after irradiation (Fig. 5E–F, Q–R). An important proportion of Ki67 positive elements were endothelial cells (data not shown). Type IV collagen labeling revealed an important rarefaction of brain vessels in the radiation target. This decrease was prominent one month after irradiation. Simultaneously, the diameter of the remaining brain vessels increased markedly. These modifications of the vascular network morphology were confined to the radiation target (Fig. 5 H–T). No similar changes were observed in the unidirectionally irradiated contralateral hemisphere (Fig. 5 G–S). Microplanar irradiation induced astrocyte activation (intensification of the GFAP labeling) in both hemispheres but was only visualized in the radiation target at D30 (Fig. 5 I–J, U–V). Finally, neuron nucleus size and shape became inhomogeneous, some nuclear fragments were detected and some neurons presented vesiculations at the nucleus/cytoplasm interface, 30 days after exposure in the radiation target, whereas no change was observed on the contralateral side (Fig. 5 K–L W–X).

### Bilateral IAMB Irradiations of Somatosensory Cortex in the GAERS Rat

**Figure 6:**
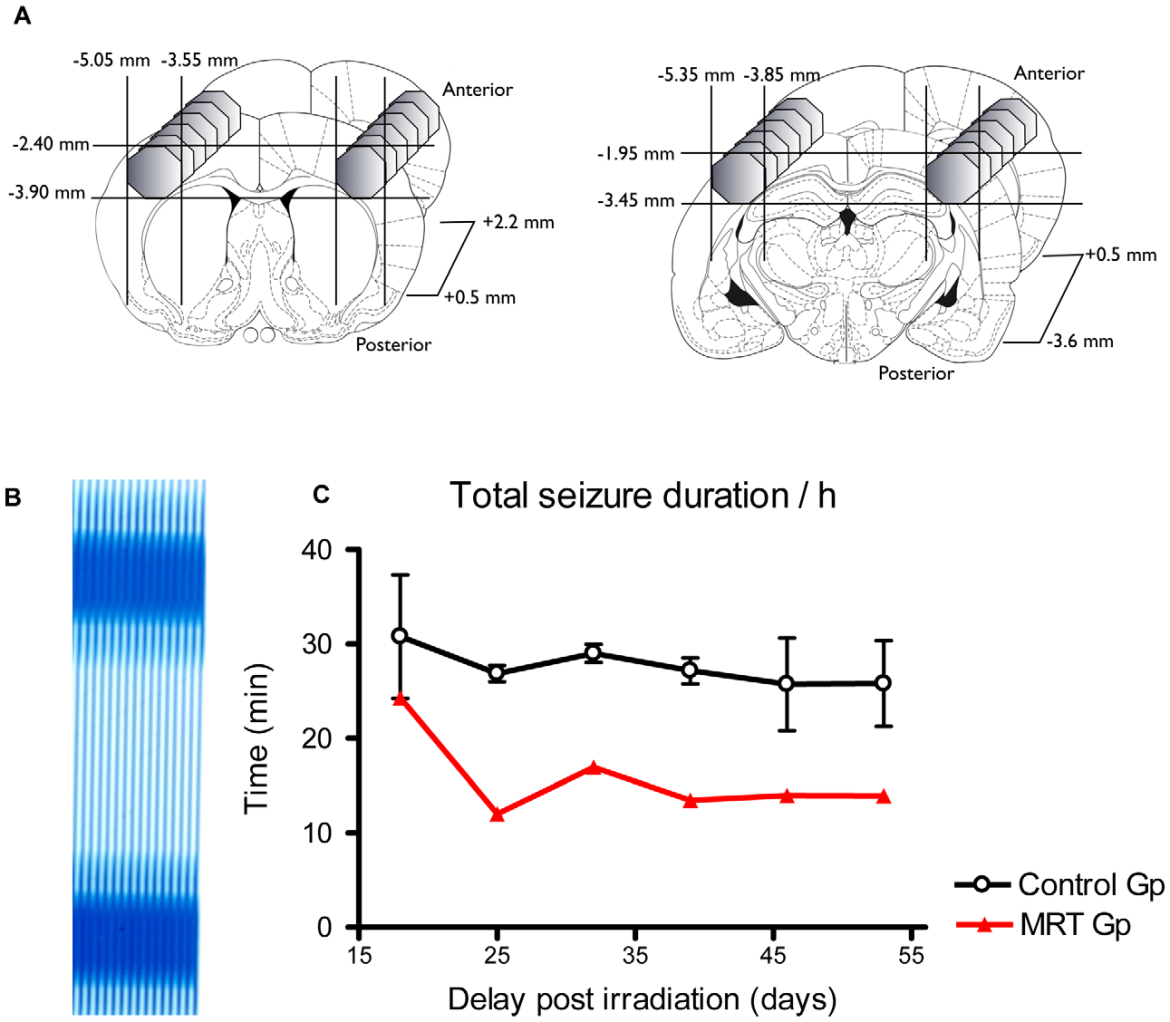
GAERS cortical irradiations and EEG follow up. A- The regions targeted in the epilepsy study were two symmetric volumes located in the two somatosensory regions of the GAERS rat cortex. Each volume consisted of two juxtaposed cylinders (left and right figures), which geometries were chosen to fit the somatosensory cortex that initiates absence seizures. Coordinates are relative to the bregma. B- Gafchromic film showing the bilateral volumes irradiated using 4 interlaced arrays of MBs with an entrance dose of 200 Gy. C- Total seizure durations measured by EEG at different times after irradiation in the control group and in the IAMB irradiated group.

To illustrate a possible practical application of IAMB irradiation, we used the Genetic Absence Epilepsy Rats from Strasbourg (GAERS) which are characterized by spontaneous bilateral and synchronous 7–9 Hz spike-wave discharges (SWDs) on the cortical EEG concomitant with behavioral arrests. Recent data showed that SWDs would be initiated in the somatosensory (S1) cortex coding for head and whiskers before their diffusion to the rest of the cortex and the ventro-basal thalamus [14], [15]. The results obtained by EEG monitoring showed that a bilateral irradiation (Fig. 6A and B) with an entrance dose of 200 Gy of the S1 cortex in GAERS reduced (∼50%) the occurrence and duration of SWDs for 2 months (Fig. 6C).

### Human-Sized Head Phantom Dosimetry

Table 1 gives a summary of the required peak entrance dose for 3 different target volumes, considering an interlacing geometry with 50 µm FWHM from either 4 ports (200 c-t-c spacing) or 8 ports (400 c-t-c spacing). The dose to be delivered to the target (2×2 mm^2^, 1×1 and 3×3 cm^2^ target) was chosen to be 100 Gy and the main factors responsible for a different dose distribution in depth (7.5 cm) are described. The Interlacement Enhancement Factor (Int. EF) corresponds to the fractional increase in radiation dose delivered to the target by 4 or 8 interlacing arrays of MBs *versus* the in-MB dose delivered by a single array of MBs. The entrance peak and valley doses for the different MB array configurations are shown, including the attenuation of the peak dose at 7.5 cm depth. The contribution from scattered photons to the peak dose becomes noticeable at larger depths and for larger MB arrays with smaller inter-beam spacings. These simulations show that whatever the irradiation field considered, peak and valley entrance doses required to deliver an homogeneous irradiation dose of 100 Gy at 7.5 cm depth are less than 300 and 10 Gy respectively.

**Table 1:** Human-sized head phantom Monte Carlo simulations.

Irradiation field	2×2 mm^2^		1×1 cm^2^		3×3 cm^2^	
Spacing	200	400	200	400	200	400
Number of ports	4	8	4	8	4	8
PVDR at 7.5 cm	51	197.5	19.7	45.9	7.9	15.9
Arbitrary dose at the interlaced region at 7.5 cm	100 Gy					
**Int. enhancement factor**	**14%**	**15%**	**22%**	**23%**	**43%**	**52%**
In beam required dose at 7.5 cm	88	87	82	81	70	66
Valley dose at 7.5 cm	1.73	0.45	4.16	1.76	8.7	4.1
Depth attenuation factor	0.30	0.30	0.30	0.30	0.32	0.31
**Entrance peak dose required**	**297**	**293**	**273**	**270**	**216**	**210**
PVDR at entrance site	71	342	38	118	22	52
**Valley dose at entrance site**	**4.2**	**0.9**	**7.1**	**2.3**	**9.8**	**4.0**

**Figure 7:**
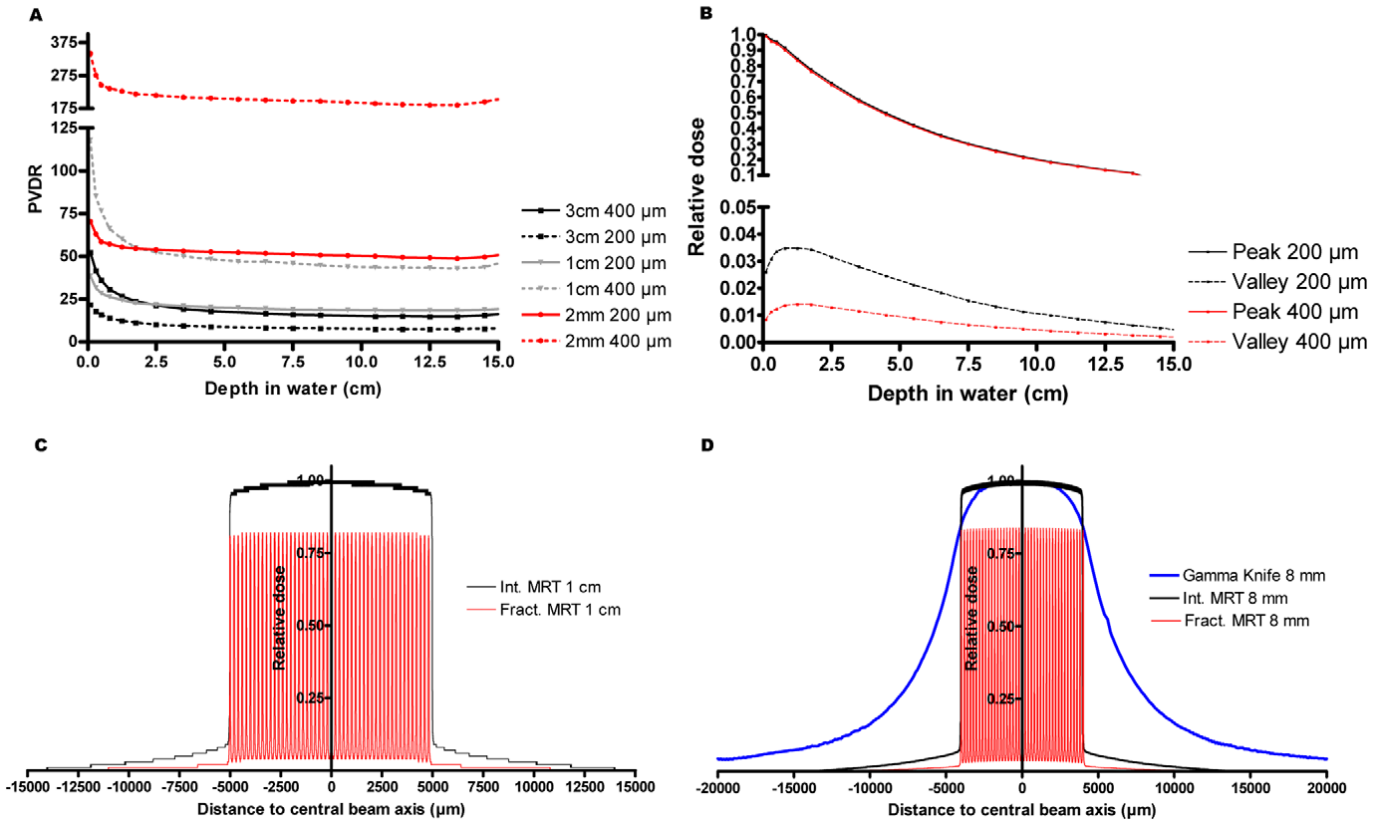
Human-sized head Monte Carlo dosimetry. A-PVDRs calculated at different depths for different quadratic irradiation fields. The field size is indicated in the legend together with the spacing between MBs). B-Peak and valley depth-dose curves in water for 200 µm and 400 µm spacing between MBs. C- Calculated dose profiles for a single MB array and for 4 interlaced arrays is shown for 1×1 cm^2^ irradiation fields. An increase in dose of 18% is measured for the dose deposited in the interlaced region (black) compared with the dose in the MB path (single array, red). D- A comparison of the lateral dose profile produced by the Leksell Gamma Knife (LGK) Perfexion® (blue) with the dose deposited by interlaced MB irradiation (black) is shown for an 8 mm wide irradiation field.

Calculated PVDRs, peak and valley doses at 7.5 cm depth and at the entrance site in a water phantom when delivering an arbitrary dose of 100 Gy to a 2×2 mm^2^, 1×1 cm^2^ or 3×3 cm^2^ target in the interlaced region (7.5 cm depth) with 50-µm wide MBs and for two different MB spacings. The interlacement enhancement factor corresponds to the increase in radiation dose in the target with respect to the in-MB dose given in a single array of MBs as represented on figure 7.

In Fig. 7A the PVDR's for the different MB configurations are plotted and show clear decrease for increasing MB array size as well as 200 µm *versus* 400 µm MB spacing. In Fig. 7B, the variation of the MB peak and valley doses with depth are shown for a 1×1 cm^2^ array of 50 µm wide MBs. The maximum peak dose is barely affected by the change in MB separation (from 400 µm to 200 µm). The valley dose on the other hand drastically increases by a factor of approximately three when the interbeam spacing is halved. In Fig. 7C the dose distribution from one port of a microbeam array is shown compared with the dose distribution in the interlaced region at identical depths. The absolute dose is increased in the cross-fired volume due to the build up of dose from electrons and X-rays scattered laterally from other microbeams. In fig. 7D, the dose produced by a MB array and the dose distribution in the interlaced region produced by 4 MB arrays is compared with the dose profile obtained with the Leksell Gamma Knife (LGK) Perfexion®. This dose profile has been calculated for a single exposure targeted to the middle of a water head phantom of diameter 16 cm. The profile has been extracted in the anterior-posterior direction of the head (the Y-direction according to LGK coordinates). The 90-10% lateral dose falloff from the non-interlaced and interlaced MB array edge occurs within a distance of 50 µm, compared with LGK where the penumbra extends over a distance of about 9 mm.

## Discussion

The results of the present study show that IAMB irradiation induced highly localized brain damage in the rat which was confined to the radiation target while the unidirectionally irradiated contralateral brain was spared. Gd-DOTA extravasation and increased ADC and T_2_W shown by MRI suggested an increase in blood brain barrier (BBB) permeability. This was associated with changes in brain vessel morphology, numerical increase in proliferating cells, monocytes/macrophages and astrocyte activation, as evidenced by immunohistochemical labeling. The effectiveness of IAMB was validated by the suppression of spontaneous seizures obtained after irradiation of the cortical focus in GAERS rats. Finally, Monte Carlo simulations for this technique performed on a human head phantom suggested the feasibility of this technique in clinical conditions. Altogether, the results obtained in this work highlighted the potential use of synchrotron light for therapeutic irradiations for human brain radiosurgery.

### Brain Tissue Tolerance to Interlaced Microbeams

The IAMB irradiation concept was first introduced in 2005 by Bräuer et *al.* [13]. It was based on the use of 2 arrays of 25 µm-wide microplanar beams (200 µm on-center distance) which generated an interlaced region where the distance between 2 MBs was 100 µm. Thus, the valley dose (dose generated by Compton effect/secondary electrons and deposited between two MBs) could be increased by a factor of 3 as compared to the unidirectionally irradiated regions. A uniform dose deposition mode has been recently used by interlacing two arrays (90°) of parallel minibeams (680 µm wide) [22]. At a 120Gy entrance dose, a “nearly perfect” lesion of 40 mm^3^ was observed whereas massive edema, which extended in the contralateral hemisphere, and important brain displacement at the target site was reported at a higher radiation dose (in-beam dose of 150Gy). In addition, the corpus callosum was displaced by about 2 mm six months after irradiation. Although the delivered dose was higher in our study (200Gy), we did not observe similar brain damages, most likely because of our use of 50 µm-wide MBs, a width linked to efficient normal tissue sparing [20], and the smaller volume of 7 mm^3^ *versus* 40 mm^3^. Indeed, as detected by MRI and immunohistochemistry, tissue damages were confined to the region where the beams interlaced, whatever the delays of observation. This is in agreement with the observation that a 4000 Gy entrance dose can be delivered to a mouse brain by a 25 µm-thin cylindrical MB without ensuing necrosis of the tissue [8]. However, the radiation dose tolerated by brain tissue decreased drastically with the increase of beam width, as a 1-mm thick cylindrical beam (entrance dose: 140 Gy) induced a macroscopic necrosis of the mouse brain tissue [8]. The effects of MB irradiation on normal brain are now well described in the literature [9], [10], [23]–[28] and our previous work showed that the use of 50-µm thick MB was a safer compromise (compared with 25 or 75 µm) between normal tissue sparing and brain tumor control for a 200 µm spacing [20]. In the present study, no damage was visualized by MRI in 4 control rats irradiated by intersecting, non-interlacing arrays which created 700Gy microplanar peak doses spaced by 200 µm on-center (valley dose of 12.5 Gy at 1 cm depth). This observation demonstrates that the extent of MB-induced damages depends to a large extent on preservation of spatial fractionation and also illustrates that IAMB irradiations through several ports would be a safe way to deliver high radiation dose to small, well-delimited brain lesions.

### Resistance of Brain Vascular Network

The radioresistance of the brain vascular network to spatially fractionated irradiations may explain the tissue tolerance to very high radiation doses. In this study, blood vessels were only damaged in the radiation target. In the contralateral hemisphere, radiogenic micro-lesions of blood vessels were rapidly repaired, in agreement with previous studies [9], [10], [29]. On the contrary, the macroscopic lesion induced in the radiation target (2 mm wide) showed a strong decrease in vessel density and an increase in vessel diameter. This was correlated with a significant increase in ADC and T_2_w signal values and Gd-DOTA extravasation at D30 after exposure. These MRI and histological features reflect vasogenic edema formation which typically follows the radiation-induced BBB disruption [30], [31]. In case of brain tumor, such an increase in tumor blood vessel permeability may facilitate delivery of chemotherapeutic agents to the lesion *via* the circulatory system. Indeed, due to the high intratumoral pressure and presence of a blood tumor barrier, systemic injection of drugs remains inefficient [32]–[34]. Because of the very high radiation dose delivered in the target region, IAMB would then (1) decrease the intra-tumoral pressure by decreasing cell density and (2) increase the amount of drugs delivered to the tumor by locally increasing the blood vessel permeability.

### Biomedical Applications of Interlaced Microbeams

Our study strongly suggests that IAMB could be considered for medical applications. The high tolerance of normal brain tissue to spatially fractionated irradiation using MBs would allow very high dose deposition in the target without impairment of radio-sensitive surrounding brain structures situated within a single array as in the case of Gamma knife [35], [36]. However, IAMB as described in the present work fulfils the criteria of a new type of radiosurgery with many advantages provided by the use of the synchrotron light.

First, the highly focused, quasi-parallel synchrotron beam (∼1 and ∼0.1 mrad horizontal and vertical divergences respectively) and the use of kilovoltage photons (50 to 350 keV) allows the conservation of the shape of the MB even in the depth of soft tissues, which is not the case when megavoltage photons are used. As shown in our study, PVDR values do not change after crossing about 1 cm of tissue and a very sharp lateral dose falloff was found for IAMB compared with a LGK Perfexion® irradiation. Using synchrotron light, only 1.5% of the radiation dose would be deposited at 10 mm from the center of the target *versus* 16.2% after LGK. The 90-10% dose falloff would be theoretically 182 fold higher in IMAPB than in LGK (0.050 *versus* 9.1 mm). MRI and histological studies clearly confirmed the high spatial selectivity of IAMB. As shown at the edge of the radiation target, tissular and DNA damages were confined, at a cellular scale, to the interlaced region. This suggests that the lateral falloff and the high dose homogeneity within the target might well improve the quality of the outcome in clinical practice. This is a critical issue as vulnerable or eloquent brain structures are often very close to the target whether it is a tumor or an epileptic focus. The submillimetric accuracy of IAMB would theoretically enable us to target specifically subdivisions of a given brain region (*e.g*., sensorimotor or limbic subdivision of the subthalamic nucleus), which is not feasible with a dose planning radiosurgical system. In patient with drug-resistant focal seizures, tiny malformations or tumors (*e.g*., cortical dysplasias, hypothalamic harmatomas) are often located in functional area and not amenable to surgical resection [37], [38]. For these patients, IAMB could reduce or even stop seizures generated by a clearly identified epileptogenic zone, as supported by our experimental proof of concept in epileptic rats.

Second, the high dose rate of synchrotron generated x-rays (∼16 000 Gy.s^−1^ at the ESRF) allows very short exposure (typically few ms) and thus avoids the smearing out of the radiation dose induced by the physiologic motion of the brain. Brain motion probably constitutes the main difficulty for the clinical transfer of IAMB. It reflects the response of the brain parenchyma, spinal cord, and CSF changes in arterial and venous pressure and volume during the cardiac cycle and was measured using different imaging techniques [39]–[43]. The γH2AX immunolabeled sections (Fig. 4), reveal that interlacing 4 arrays of MBs was not hindered by brain motion in rats. However, brain motion in rat is likely to be more limited than in the human head, where brain motion ranges from 50 µm in the occipital, frontal and parietal lobes to 100 µm in the hypothalamus [42], with a maximum (184 µm) in the pons [44]. Most of these values are in the range of the size and spacing of the MBs in IAMB. This might alter the efficiency of the treatment by modifying the irradiation geometry. However, brain motion has a maximum velocity of 2 mm.s^−1^, a few milliseconds after the R wave [45]. In addition, every brain structure has been reported to go back with a µm accuracy to its initial position after complete heart cycle, [42]. Therefore, heart-gated synchronized irradiations will be required in IAMB for clinical use and the design of the ID17 beamline [16] and a fast shutter [46] might facilitate this implementation. The very high dose rate of the source would considerably reduce the exposition times giving the possibility to treat several lesions in the same patients in a limited time. This is a critical feature when treating multiple tumor metastases in the brain [47], [48].

Finally, the low energy of the x-ray photons used in IAMB might constitute a severe disadvantage, when compared with megavoltage photons of conventional radiotherapy. However, the 50–350 keV white beam produced by the synchrotron preserves an adequate PVDR; the penetration of these photons permits delivery of important radiation doses deeply in soft tissues. In our study, the dose falloff is only 30% of the nominal dose at 7.5 cm depth. In addition, our irradiation geometry compensates this decrease. Indeed, the dose contribution of the lateral diffusion of the secondary electrons and photons increases the radiation dose delivery in the radiation target compared with the individual array. This so called “Interlacement Enhancement Factor (Int.EF)” increases with the size of the irradiation field and, to smaller extent with MB spacing, up to 50% for a 3×3 cm^2^ irradiation field. Despite the fact that the PVDR values and valley doses become respectively lower and higher with the increase in field size, the Int.EF allows a significant decrease in the entrance doses (peak and valley). Thus, for a 100 Gy irradiation of a 27 cm^3^ target located at 7.5 cm depth, valley and peak doses in each arrays of MBs are less than 10 and 300 Gy, which are considered tolerable for normal brain tissue [9], [10], [25], [26], [28], [49]–[51]. By increasing the number of ports from 4 to 8, valley doses deposited in each array of MBs can be reduced to about 4 Gy or less. The reduction of the radiation-induced edema observed after MB exposure [9], [10] would be very critical for thalamotomy [52]. Finally, for patients with arteriovenous malformations where lesion's size represents a serious limitation, the fact that high-radiation dose could be delivered using IAMB without inducing severe edema should be extremely helpful [53].

In conclusion, we show here that IAMB allows a discrete high dose deposition in a given brain region and induces in confined damages, sparing surrounding tissues. This new irradiation method could be useful for any brain disease where extremely precise focal tissue destruction is required. As a proof of principle, bilateral IAMB irradiation of the somatosensory cortex, shown to initiate spontaneous epileptic seizures in GAERS rats, significantly reduced the occurrence and duration of electroencephalographic spike wave discharges. Finally, the Monte-Carlo simulations suggest that synchrotron-generated low energy x-rays are a promising tool for high uniform dose delivery in human brains with an extraordinarily sharp dose falloff. We suggest that most applications of LGK radiosurgery are technically transposable by adaptations of synchrotron x-rays for IAMB irradiations in stereotactic conditions with optimal conformality.
